# A mixed-methods systematic review of mental health activities among urban left-behind children in mainland China

**DOI:** 10.3389/fpubh.2024.1431996

**Published:** 2025-01-20

**Authors:** Siyu Long, Zainal Bin Madon, Noris Mohd Norowi, Mei Foong Ang

**Affiliations:** ^1^Department of Music, Faculty of Human Ecology, Universiti Putra Malaysia, Serdang, Malaysia; ^2^Industry Polytechnic College, Jiangxi, China; ^3^Department of Human Development and Family Studies, Faculty of Human Ecology, Universiti Putra Malaysia, Serdang, Malaysia; ^4^Department of Multimedia, Faculty of Computer Science and Information Technology, Universiti Putra Malaysia, Serdang, Malaysia

**Keywords:** urban left-behind children, mental health activities, barriers, facilitators, mainland China

## Abstract

**Introduction:**

The mental health of left-behind children has garnered attention from Chinese scholars in recent years. Although several interventions have been implemented to address these children’s mental health in urban areas, a gap remains in understanding the types of interventions, their effectiveness, and the factors that act as barriers or facilitators during the implementation process.

**Methods:**

A mixed methods systematic review informed by JBI methodology. Researchers conducted a comprehensive search of databases in both English and Chinese, covering the years 2005 to 2023. The initial search took place in January 2024 and was updated in March 2024. This study includes all studies results available up to December 31, 2023. The protocol has been registered on PROSPERO (CRD42023384078) and includes 14 studies in the review.

**Results:**

The activity categories included group psychological activities, individual family activities and multiple formats services. Three barriers to implementation emerged: social workers, activities and parents. The facilitators were parents and activity design.

**Conclusion:**

This review revealed that some studies suffered from poor data collection methods and data quality. Studies on services for mental health in urban left-behind children requires methodologically robust study designs for broader dissemination and rigorous evaluation.

**Systematic review registration:**

PROSPERO, CRD42023384078, available from: https://www.crd.york.ac.uk/prospero/display_record.php?ID=CRD42023384078.

## Introduction

Mental health issues are increasingly affecting human life. The COVID-19 pandemic significantly exacerbated global mental disorder prevalence (1–3). Without early identification and intervention, mental illness can result in mental disorders, psychosocial disabilities, and other mental states. Substantial distress, impaired functioning, or self-harm risk characterise such mental states.

In 2019, nearly one billion people worldwide had diagnosable mental disorders, of which approximately 14% was adolescents (4). In China, mental health issues affected an estimated 173 million people (5), of which approximately 30 million were children aged <17 years old (6). Children’s mental health issues affect their cognitive, emotional, and behavioral performance (7–9) and substantially influence their adult lives (10, 11).

In China, the numerous left-behind children (LBC) are vulnerable to mental health issues (12, 13). With China’s rapid economic growth and accelerated urbanization, many workers from rural and less developed areas have been attracted to seek opportunities in developed cities (14). This trend has led to a dramatic increase in China’s migrant population, which soared from 6.6 million in 1982 to 245 million in 2017 (15). Due to a variety of complex factors, these migrants often cannot bring their children with them, leaving the children in their hometowns under the care of grandparents, relatives, friends, community organisations, or schools. These children are commonly referred to as LBC (16). By the end of 2020, approximately 66 million children in China were left-behind, constituting 22 percent of the national child population (17). According to China’s household registration system, the National Bureau of Statistics of China et al. (17) define rural left-behind children (RLBC), as those whose household registration is in rural areas and who reside there, while urban left-behind children (ULBC) refers to those whose household registration is in urban areas and who reside there.

Although both RLBC and ULBC experience separation from their parents and share the characteristic of being left-behind, significant disparities exist in their circumstances, including living conditions (18), family backgrounds (19), parental contact rates (20), and mental health statuses (15, 21). The most pronounced difference is the environments in which they reside: ULBC live in urban areas (18), whereas RLBC are situated in rural settings (22). Compared to RLBC families, ULBC families generally have greater financial stability and better access to educational and social welfare resources (23). However, ULBC appear to be more susceptible to mental health issues than RLBC. A survey involving over 5,000 students from 18 randomly selected urban and rural schools in Anhui Province revealed that ULBC exhibited more emotional symptoms and hyperactivity than RLBC (15). Additionally, Zhang et al. (21) compared personality traits between ULBC and RLBC, finding that ULBC displayed lower levels of emotional stability, resilience, collective consciousness, and empathy.

ULBC numbers are large and increasing. The National Bureau of Statistics for China Population Census reported approximately 25.17 million ULBC in 2020 (17). This number implies a 30% increase compared to the number in 2010 (24). Concurrently, the number of implicit ULBC is gradually increasing in Chinese cities. Implicit ULBC refers to a situation where both parents and the child live in the city, yet one or both parents have busy work schedules that make it difficult to find time to spend with the child. This lack of parental companionship is similar to ULBC; therefore, there children are referred to as implicit ULBC (25). Implicit ULBC might result from increased commuting time, work stress, and living costs, which occur more frequently in the urban rather than rural setting.

Despite parents’ efforts to improve their economic status in order to better support their children, prolonged separation from their parents exposes ULBC to significant mental health issuers. Over the past two decades, scholars have developed a range of mental health activities aimed at addressing the mental health needs of ULBC. Although studies into the mental health of ULBC is on the rise, there has yet to be a comprehensive academic review of mental health activities for this group.

To date, this study may be the first systematic literature review examining mental health activities for ULBC. By employing a mixed-methods systematic review, this study comprehensively evaluates the types, effects, barriers, and facilitators of mental health service implementation for ULBC, offering valuable references and improvement strategies for future initiatives. Additionally, the study provides critical insights for policymakers, researchers, and funding agencies regarding existing gaps and boundedness in research breadth. The study questions for this review are as follows:

1. What types of activities were developed to improve the mental health of ULBC, and how effective were these activities?
2. What were the barriers to and facilitators of implementing these activities?

## Method

To obtain a broader range of evidence, this study adhered to the Preferred Reporting Items for Systematic Reviews and Meta-Analyses (PRISMA) guidelines proposed by Stern et al. (26). The protocol was registered with PROSPERO (CRD42023384078). Evidence was synthesised using the convergent integrated approach (26) according to the Joanna Briggs Institute (JBI) methodology for mixed-methods reviews.

### Search strategy

Electronic databases were searched for relevant studies published between 2005 and 31 December 2023. To comprehensively search for relevant studies on the mental health activities of ULBC, this review accessed multiple key databases in both English and Chinese. The English-language literature was searched using PubMed, CINAHL Complete, PsychINFO, ScienceDirect, Web of Science, Scopus, ProQuest Dissertation and Theses, Connected Papers, and Google Scholar. Chinese-language study was searched using the China National Knowledge Infrastructure (CNKI) database, the Wanfang Data Knowledge Service Platform, and the Wipu Chinese Science and Technology Journal Database. The initial search was conducted in January 2024, with the search results updated in March 2024. All studies results from 2005 to 31 December 2023 were included.

### Inclusion and exclusion criteria

Inclusion and exclusion criteria were specified using the PICo framework (Population, phenomena of Interest and Context). According to the United Nations Convention on the Rights of the Child, children are persons under the age of 18 years, i.e., between 0 and 17 years of ages (16). Therefore, the participants included in the study were ULBC aged 0–17 years, males and females. ULBC aged >17 years were excluded from the study. Studies in which participants were Chinese LBC and RLBC mental health were excluded, yet their reference lists were reviewed for additional scoping reviews.

This review examines not only the types and effectiveness of mental health activities, but also the barriers and facilitators involved in their implementation. To this end, the study incorporates a broad range of evidence, including qualitative, quantitative, and mixed-method studies. In most Chinese literature, qualitative studies typically use the term “mental health service activities,” whereas quantitative studies more commonly refer to “mental health intervention activities.” Therefore, the term “mental health activities” has been standardised to encompass both types.

The phenomena of interest (interventions) are mental health services or intervention activities implemented by ULBC. Mental health activities are defined as those that utilise psychological principles and techniques to assist in improving an individuals’ emotional state, behavioral patterns, and health-related psychological conditions through various means (27). Therefore, included studies were required to report the mental health status of ULBC following the receipt of mental health activities. Studies that did not report the mental health status of ULBC after receiving these services or interventions were excluded.

Since Hong Kong, Macau, and Taiwan all practice a capitalist system, they differ from the socialist system in mainland China. As those regions are more economically developed and possess more public resources, their mental health services developed earlier, and their systems are relatively mature. Consequently, the characteristics of mental health issues among ULBC in these regions may vary from those in mainland China. Therefore, studies from these regions were excluded from this review in order to more precise understand the characteristics of mental health activities of ULBC in mainland China, enhancing the relevance and practical application of the findings.

Only papers published in English or Chinese between 2005 and December 2023 were included. The studies entailed undergraduate theses, master’s theses, doctoral dissertations, and conference reports. Articles published before 2005 were excluded because the issue of left-behind children gained scholarly attention after the China’s Ministry of Education issued an educational directive in 2005 (28).

### Study selection

Long and Zainal conducted the title and abstract eligibility screening. The identified articles were collated in Zotero and duplicates were removed. Long and Zainal recorded the reasons for exclusion, which were independently validated by Noris and Ang. A study that only discussed the ULBC’s mental health condition without providing ULBC mental health services or interventions was excluded. The eligible studies underwent full-text screening, with disagreements resolved through discussion between the four researchers to achieve consensus.

### Quality appraisal

The overall quality of the included studies was assessed using Mixed Methods Appraisal Tool (MMAT) version 2018 (29). The MMAT was used as it is a more reliable and valid quality assessment tool for critical evaluation of qualitative, quantitative, and mixed-method studies (30). Long and Zainal rated each study independently. Disagreements were resolved through discussion with Noris and Ang. All dimensions were divided into “yes,” “no,” or “undiscerned,” with “undiscerned” selected when the study provided insufficient information (see Table 1).

**Table 1:** Quality appraisal of included studies using the Mixed Methods Appraisal Tool (MMAT).

Authors, year	Screening		Qualitative studies					Quantitative randomisation controlled trials					Quantitative non-randomisation studies					Score
	S1	S2	1.1	1.2	1.3	1.4	1.5	2.1	2.2	2.3	2.4	2.5	3.1	3.2	3.3	3.4	3.5	
Song (43)	Yes	Yes	Yes	Yes	No	No	Yes											60%
Wu (41)	Yes	Yes	Yes	Yes	Yes	Yes	Yes											100%
Jiang (38)	Yes	Yes	Yes	Yes	Yes	Yes	No											80%
Yang (40)	Yes	Yes	Yes	Yes	Yes	Yes	Yes											100%
Sun (39)	Yes	Yes	Yes	Yes	Yes	Yes	Yes											100%
Ren (44)	Yes	Yes	Yes	Yes	No	Yes	No											60%
Zhang (42)	Yes	Yes	Yes	Yes	Yes	Yes	Yes											100%
Zhang (37)	Yes	Yes	Yes	Yes	No	No	Yes											60%
Li (45)	Yes	Yes	Yes	No	No	No	No											20%
Ge et al. (32)	Yes	Yes						Yes	Yes	Yes	Undiscerned	Yes						80%
Hu (33)	Yes	Yes						Yes	Yes	Yes	Undiscerned	Yes						80%
Yi and Wang (35)	Yes	Yes											Yes	Yes	Yes	Undiscerned	Undiscerned	60%
Liu (34)	Yes	Yes											Yes	Yes	Yes	Yes	Undiscerned	80%
Zhang (36)	Yes	Yes											Yes	Yes	Yes	Yes	Undiscerned	80%

Screening questions (for all studies). S1: Are there clear research questions? S2: Do the collected data allow to address the research questions? Qualitative. 1.1. Is the qualitative approach appropriate to answer the research question? 1.2. Are the qualitative data collection methods adequate to address the research question? 1.3. Are the findings adequately derived from the data? 1.4. Is the interpretation of results sufficiently substantiated by data? 1.5. Is there coherence between qualitative data sources, collection, analysis and interpretation? Quantitative randomisation controlled trials. 2.1. Is randomisation appropriately performed? 2.2. Are the groups comparable at baseline? 2.3. Are there complete outcome data? 2.4. Are outcome assessors blinded to the intervention provided? 2.5 Did the participants adhere to the assigned intervention? Quantitative non-randomisation. 3.1. Are the participants representative of the target population? 3.2. Are measurements appropriate regarding both the outcome and intervention (or exposure)? 3.3. Are there complete outcome data? 3.4. Are the confounders accounted for in the design and analysis? 3.5. During the study period, is the intervention administered (or exposure occurred) as intended.

### Data extraction

Data were extracted from each included article (aim, paradigm, setting, sample and participant characteristics, mental health activity type, implementation details, data collection methods, results, barriers and facilitators) using MMAT tools. Long and Zainal extracted the data, which were independently validated by Noris and Ang. Inaccuracies or discrepancies were resolved through discussion.

### Data synthesis

The data were synthesised based on the JBI methodology. This review followed the JBI convergent integrated mixed-methods approach to synthesise and integrate both qualitative and quantitative data. The convergent integrated approach allows for the conversion of qualitative data into numerical values, or quantitative data into themes, categories, typologies, or narratives. To enhance the accuracy, the researchers followed the recommendation of Sten et al. (26) to paraphrase the entire quantitative data into concise sentences, which were then aggregated and combined with the qualitative data extracted from the study. Subsequently, the researchers employed the thematic analysis to summarize and categorise the combined data. The six-step process proposed by Braun and Clarke (31) was followed: (1) familiarisation with the materials; (2) coding; (3) generation of themes; (4) Theme review; (5) defining and naming themes; and (6) reporting of results. To enhance the accuracy and objectivity of the analyses, the two researchers independently performed the coding tasks. Long used NVivo 11 software for coding, while Zainal conducted manual coding. After coding, they engaged in cross-validation and in-depth discussions to ensure the comprehensiveness and accuracy of the code assignments. The codes were then further categorised into descriptive themes. In cases where identifying themes posed challenges, the researchers revisited the full text and engaged in exhaustive discussions until a consensus was reached. Finally, the themes, categories, and corresponding quotes were reviewed by Noris and Ang to ensure they accurately reflected the content of the original data.

## Results

### Article review

**Figure 1:**
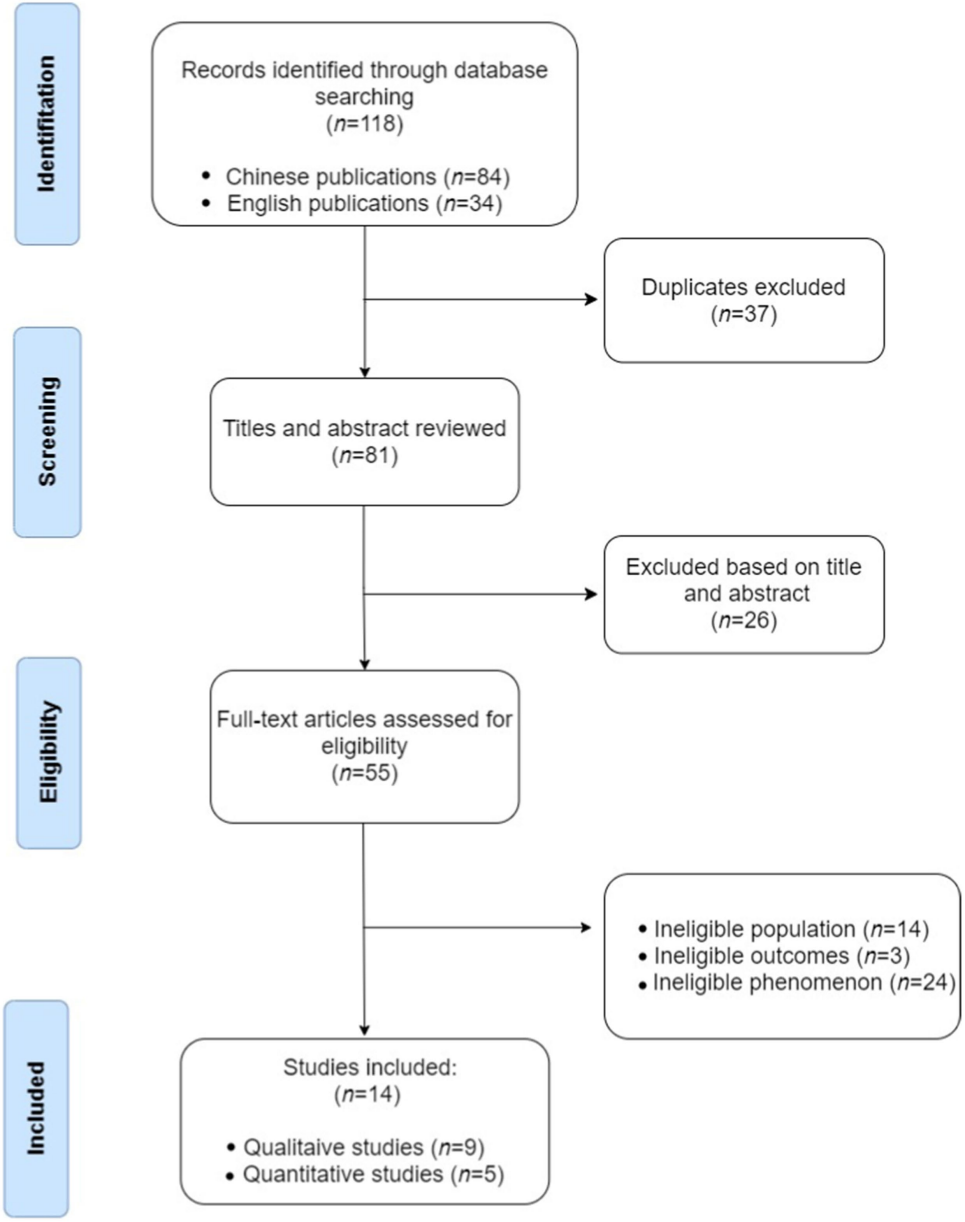
Flow diagram of search process.

The initial search conducted in March 2023 yielded 118 potentially relevant studies (84 studies in Chinese and 34 studies in English). Duplicate data removal and relevance screening resulted in 81 studies meeting the title- and abstract-based eligibility criteria. The title and abstract screening excluded 26 studies on LBC. Consequently, 55 studies were retained for further screening. The literature review focused on the participation of ULBC in mental health studies. It excluded 24 studies that merely described the current state of ULBC mental health without intervention, 14 that failed to meet age or location criteria, and three that lacked specific inquiries aimed at improving mental health. Ultimately, 14 articles were selected for systematic evaluation. Figure 1 illustrates the flowchart for this review.

### Methodological quality

Among the included quantitative studies, Ge et al. (32) and Hu (33) used randomisation controlled trials (RCT) of interventions for ULBC mental health. As neither study reported whether the outcome assessors were blinded to the intervention, they were labelled “undiscerned.” Three studies (34–36) used non-randomisation works (before-after study of the same patient) to intervene in ULBC mental health. As the three studies did not report on the study design and whether confounders were considered in the analyses, they were also labelled “undiscerned.” Furthermore, Yi and Wang (35) did not report whether the intended intervention was implemented; therefore, the study was also labelled “undiscerned”.

The other nine studies conducted qualitative study. These studies assessed ULBC mental health using interviews and observations. The included studies measured ULBC mental health outcomes using scales, such as the Chinese adaptation of the Interpersonal Relationships Rating Scale (IRRS) (37), the parent–child relationship diagnostic test (PCRT), and the Child–Parent Relationship Self-Rating Scale (CPRSRS) (38–40).

Four qualitative studies (39–42) had high overall methodological quality. The four studies were awarded “yes” for all five methodological quality criteria, which yielded a score of 100%. This result indicated appropriate reporting of the methodology and findings. Song (43) and Zhang (37) only reported data from observing the participants. Therefore, their data were deemed insufficient to support their results. Jiang (38) used different parent–child relationship self-assessment scales before and after the mental health intervention. Consequently, the data and interpretation of the qualitative information were inconsistent.

Ren (44) administered a mental health questionnaire to the participants only after the mental health service and interviewed the participants’ surrogate parents, community residents, and social workers. Thus, their data were considered insufficient to refine the results. Furthermore, their qualitative study data, analysis, and interpretation were inconsistent.

Li (45) gained a more comprehensive understanding of the participants’ mental health by conducting pre-service interviews with the participants and the participants’ grandparents, parents, and teachers. Nonetheless, the study data was only based on the researcher’s observation that the mental health service enhanced the participants’ mental health. Thus, the data collection methods, interpretation, and consistency and conclusions were inadequate. Consequently, the aforementioned sections were labelled “undiscerned”.

### Characteristics of the included studies

Fourteen of the included studies were published in Chinese. Of these, three studies were published in journals (32, 35, 37) and the remaining studies were published as thesis dissertations. The 14 studies were conducted by Chinese academicians with sample sizes of 1–65 ULBC. The mental health activity durations were 7–15 weeks. Table 2 details the aims, paradigms, settings, sample and participant characteristics, data collection methods, and results of each included study.

**Table 2:** Characteristics of the included studies.

Authors (year)	Article type	Aim	Paradigm	Setting	Sample	Participant characteristics	Data collection methods	Results
Song (43)	Thesis	To describe community activity experiences that helped improve ULBC social integration	Qualitative	Q City	*n* = 26 (age not reported)	Explicit (intergenerational and single-parent guardianship) and implicit left-behind	Observation, semi-structured interviews	ULBC’s psychological well-being improves as they begin to empathise with their parents, leading to increased interaction, alongside noticeable progress in their learning. Additionally, ULBC parents have achieved a certain proficiency in parent–child communication.
Wu (41)	Thesis	To explore the influence of group activities on ULBC psychological resilience	Qualitative	Wuhan (central)	*n* = 6 (6–8 years old)	Explicit left-behind (intergenerational, single-parent, kinship guardianship)	Questionnaire, observation, interview	ULBC are increasingly willing to take the initiative in expressing themselves, demonstrating confidence in their abilities and in life, and holding the belief that they can attain their goals through diligence. When faced with challenges, they are open to seeking assistance from those around them.
Jiang (38)	Thesis	To explore the parent–child group’s experience of enhancing ULBC parent–child relationships	Qualitative	Chengdu (western)	*n* = 6 (aged 6–8 years old)	Not reported	Questionnaires (self-administered parent–child relationship questionnaire and Chinese adaptation of the PCRT), observation.	Six participating families had significant improvements in their parent–child relationships.
Yang (40)	Thesis	To describe the experience of improved parent–child relationships in grandparent-raised ULBC	Qualitative	Chongqing (western)	*n* = 1 (10 years old)	Explicit left-behind (intergenerational guardianship)	Questionnaire (CPRSRS), observation, interview	The ULBC showed a heightened willingness to share aspects of their learning and life with their parents, leading to a notable enhancement in their relationship.
Sun (39)	Thesis	To investigate the experience of social work services in improving the ULBC parent–child relationship	Qualitative	Nanjing (eastern)	*n* = 1 (10 years old)	Explicit left-behind (intergenerational guardianship)	Questionnaire (Parent–Child Relationship Self-Assessment Scale [PCRSAS]), observation, interview	The improvement not only enhances the relationship between ULBC and their parents but also fosters their self-confidence and interpersonal skills.
Ren (44)	Thesis	To describe experiences of community activities that helped ULBC improve mental health	Qualitative	Y City	*n* = 10 (8–12 years old)	Not reported	Interview, questionnaires.	ULBC have experienced a marked improvement in mood, a sense of happiness, and have made new friends
Zhang (42)	Thesis	To explore the experience of social work services in improving ULBC mental health	Qualitative	J City	*n* = 1 (7 years old)	Explicit left-behind (intergenerational guardianship)	Interview, observation	Parent–child relationship, interpersonal skills, and attention improved significantly; lying behavior decreased significantly. Begins to consider the feelings of others and is less self-centred.
Zhang (37)	Article	To record the effect of a group sandplay on ULBC interpersonal skills	Qualitative	Unknown	*n* = 6 (Grade 7)	Not reported	Questionnaires (Chinese adaptation of IRRS), interviews, observation	Interpersonal skills improved significantly
Yi and Wang (35)	Article	To examine the influence of drawing activities on ULBC mental health	Quantitative	Chongqing (western)	*n* = 65 (7–16 years old)	Not reported	Questionnaire (Chinese adaptation of the Mental Health Test [MHT])	Emotional management, self-concept, and social function improved significantly
Ge et al. (32)	Article	To examine the effect of group psychodrama on ULBC Internet addiction	Quantitative	Chongqing (western)	*n* = 24 (14 years old with an Internet addiction)	Explicit left-behind (intergenerational guardianship)	Interviews, questionnaires (Chinese Internet Addiction Scale [CIAS] and Chinese adaptation of the Social Avoidance and Distress Scale [SADS])	Notable decline in the time and frequency of ULBC’s internet use, leading to a more rational allocation of time for life and study. Social avoidance behaviors decreased, while interpersonal interactions became more active.
Hu (33)	Thesis	To examine the effects of DMT on ULBC deviant behavior	Quantitative	Changsha (central)	*n* = 24 (6–11 years old with deviant behavior)	Explicit left-behind (intergenerational, single-parent, kinship guardianship)	Questionnaires (Chinese adaptation of the Child Behavior Checklist [CBCL] and Rutter’s Children’s Behavior Questionnaire scale [CBQ]), interviews, observation	Significant reduction in ULBC’s deviant behavior, especially on antisocial and neurotic behavior.
Li (45)	Thesis	To improve learning motivation and attitude, and reduce learning pressure	Qualitative	Haikou (southern)	*n* = 10 (Grade 7)	Explicit left-behind (intergenerational guardianship)	Interviews, observations	Relationships between ULBC and their teachers became more harmonious, with notable improvements in satisfaction levels.
Liu (34)	Thesis	To enhance ULBC self-efficacy	Quantitative	Tianjin (northern)	*n* = 5 (8–10 years old)	Explicit left-behind (intergenerational guardianship)	Interviews, questionnaires (Chinese adaptation of the learning self-efficacy scale)	Group work significantly enhanced ULBC’s self-efficacy.
Zhang (36)	Thesis	To improve ULBC resilience	Quantitative	Shenzhen (southern)	*n* = 8 (10–14 years old)	Not reported	Interviews, questionnaires (Chinese adaptation of the adolescent psychological elasticity sexuality scale)	Joint coordination among family, school, and community effectively raised resilience levels in ULBC.

### Activity types and effect (influence)

The mental health activities included individual family activities, group psychological activities, and multi-format activities. The included studies demonstrated that these activities positively affected ULBC mental health. Table 3 summarises the types of activities, implementation details, and effects or influences.

**Table 3:** Summary of the types of activities, implementation details and effects or influences.

Mental health activity type	Implementation details	Effect/influence
Group psychological	Seven 50-min activities distributed over seven weeks. Games for groups (name solitaire, resilience short stories, hug game); coaching ULBC to express their daily challenges and suggest answers; coaching on using networks to overcome obstacles and enhance confidence (41).	Self-identity, sense of purpose, and social competence gradually increased
	Six activities once a week for six weeks. Parent–child games; training parent–child communication skills; guiding ULBC to develop a sense of cooperation; guiding ULBC to express emotions rationally (38).	Parent–child relationship improved significantly
	Four group sandplay sessions (chaos, vitality, group reflection, spiritual home) (37).	Interpersonal skills improved significantly
	Two 90–120-min sessions per week for 15 weeks. Five themes: doodling, portraits, scenes, feelings, group painting (35).	Mental health levels improved significantly overall, with particularly significant changes in anxiety, loneliness tendencies and impulsive tendencies.
	Two hours weekly for five weeks. Five modules, five warm-up activities, 12 psychological sketches. Warm-up activity; psychological sketches (given a theme, no pre-set script, ULBC can play with it); group sharing and discussion (ULBC expressed feelings and experiences) (32).	Internet addiction and social avoidance symptoms decreased significantly
	Twelve weeks of three 60-min sessions. Group games (self-introduction, breathing exercises, sarong, elastic step, mirroring); art games (breathing exercises, mirroring, imitation, writing, drawing, group discussion); group games (listening, cooperation, dance imitation) (33).	Antisocial and neurotic behaviors decreased significantly
	Six weeks of group activities: warm-up activities (learning to blow), discussion (is learning pain or pleasure), observation and comprehension (watching videos), discussion (how to focus the mind and overcome the fear of learning), communication skills (you draw and I guess), reflections (45).	Relationships between ULBC and teachers became more harmonious and dissatisfaction with teachers improved
	Four-week group activity consisting of seven 45–60-min sessions each, two sessions per week. The activity included self-evaluation, sharing and encouragement, learning to communicate with others, managing emotions, learning from role models, and discussion (34).	ULBC self-efficacy increased significantly
	A total of seven weeks, one session per week, approximately one hour per activity. The activity included getting to know each other and yourself, identifying negative emotions, increasing group interaction, learning to communicate, using nearby resources for aid, reflection (36).	ULBC resilience was enhanced
Individual family	Three phases of activities spanning three months. Establishing a relationship through home visits; conversing with ULBC, parents, and grandparents in turn; assisting in enhancing communication between grandparents and ULBC and between grandparents and parents; supporting family role repositioning and resolving intergenerational conflicts; assisting ULBC in regaining self-awareness (40).	Parent–child relationship improved significantly
	Activities divided into three periods of three-month durations. Establishing a rapport during home visits, identifying parent–child issues through interviews, creating a social work intervention model, advising ULBC to improve engagement with parents (letter-writing, film-watching, teaching parent–child communication skills, role simulation, classroom tutoring, group outings) (39).	Parent–child relationship, self-confidence, and interpersonal skills improved significantly
	Three-hour interventions for Week 1, then once or twice a month for the following two months for a total of eight interventions over three months. Establishing a connection, predicting and diagnosing ULBC psychology through observations and interviews, creating counselling goals and plans, offering interventions (interactive games, guided emotional release and expression, increased communication with parents, social games, increased communication with grandparent and teachers, parent–child communication groups) (42).	Parent–child relationship, interpersonal skills, and attention improved significantly; lying behavior decreased significantly
Multiple formats	Parent–child games (self-introduction, card methods), children’s group activities (craft projects), establishing a club system (including leader elections, show-of-hands voting, expressing feelings, role division), developing a reward system (point mechanisms and redemption platforms for point exchange), organising community service events (public performances and elderly home visits) (43).	Significantly improved social integration
	Two organisations aided the ULBC (volunteer organisation and children’s mutual aid society); three Internet platforms were provided to the ULBC (volunteer interaction platform, LBC growth platform, society cooperation platform). Partnering with surrogate parents, establishing a points mechanism, leader selection and training, establishing clubs, creating club charters, organising charity activity participation (environmental protection and tree planting activities) (44).	Happiness improved significantly

#### Individual family activities

Individual family activities involve social workers providing personalised counselling, guidance, and support by establishing a good working relationship with the individual or family. Such services also involve adopting different approaches to aid ULBC in resolving issues and changing undesirable behaviors. Three studies improved the relationships between the ULBC and their parents using individual family activities (39, 40, 42). The aforementioned studies involved small samples comprising only one person. The researchers were social workers who used an action research approach to access the ULBC families. The intervention involved one-on-one interviews and family activities with ULBC and family members to improve the relationships between ULBC and their parents.

The three studies involved a three-month service period with relatively similar service activities. For example, the social workers communicated with the ULBC, their families, or guardians and increased communication frequency between the ULBC and their parents or guardians, taught parent–child communication skills, and organised parent–child games. These studies reported significantly improved relationships between the ULBC and their families following the activities and improved ULBC self-perception bias (40), self-confidence (39), and lying behavior (42).

#### Group psychological activities

Group psychological activities involve social workers organising group activities for ULBC with the same background, needs, or problems. The interaction and communication among group members can resolve the individual ULBC mental health issues.

Nine included studies used group organisation and group activities to aid ULBC with mental health conditions or problematic behaviors, such as improving ULBC resilience (36, 41), self-efficacy (34), loneliness and impulsivity (35), anhedonia (45), relationship disorders (37), parent–child relationships (38), social avoidance and Internet addictive behaviors (32), and behavioral deviance (33). The intervention activities were diverse and included sand play, drawing, psychodrama, dance movement therapy (DMT), and group games.

In the following studies, the interventions spanned 5–15 weeks. One study only reported the number of interventions but not the intervention duration (37). The maximum sample size was 65 (35) and the minimum was six (38, 41). Three studies conducted a return assessment after the group activity (32, 33, 37). All the studies reported improved ULBC mental health outcomes.

#### Multi-format activities

Multi-format activities involve social workers obtaining social support from multiple sources through various means and approaches. The support includes community, school, social media, and network resources for social work to address social issues. Two studies used multi-format activities to improve general ULBC mental health (43, 44). The services united the local community, schools, volunteers, ULBC, and their families to improve the ULBC’s mental health issues.

Song (43) provided several services to 26 ULBC to improve their social inclusion. The services included (1) social workers and volunteers organising group games and psychological counselling for ULBC and their parents; (2) organising ULBC group activities; (3) aiding the ULBC in increasing communication frequency with their parents; (4) establishing a ULBC mutual support society and developing a society system; and (5) planning and encouraging ULBC participation in charity activities. Nonetheless, the researchers did not report the ULBC’s specific ages.

Ren (44) reported that the ULBC’s mental health conditions were more complex and comprehensive. A multi-faceted guarantee was guaranteed for ULBC mental health by including two organisations (volunteer organisation and children’s mutual aid society) and three internet platforms (volunteer interaction platform, LBC growth platform, and societal cooperation platform) compared to Song (43). Both studies reported a positive effect on ULBC mental health.

### Barriers and facilitators to ULBC’s physical activity

A total of five themes were identified in ULBC mental health services or interventions activities, including three barriers and two facilitators. The barriers to implementation in the included studies revealed three themes: social workers, activities, and parents. Three studies did not report barriers to implementation (33, 35, 37). Two themes emerged from the facilitators of implementation: parents and activities. It is worth noting that parents are both a barrier and facilitator. Table 4 summarises the barriers and facilitators in mental health services or intervention activities.

**Table 4:** Summary of barriers to and facilitators of ULBC’s physical activity.

Barrier/facilitator	Theme	Category	Example excerpts and quote(s)
Barrier	Social workers	Expertise	Some social workers have not systematically studied social service theoretical knowledge; therefore, they do not have a good understanding of professional social work knowledge and concepts (41, 43).
		Practical experience	Some social workers lack technical training for multi-format activities, which results in a lack of articulation between activities during actual social work (44). While some social workers have relatively rich theoretical expertise, they lack practical service experience when addressing activity processes and unexpected situations (39, 40).
	Activity	Implementation process	The uncontrollable nature of the outdoors (44), the challenge of coordinating participants’ time and views in multi-format activities (43), the emotional engagement of psychodrama (32).
		Implementation approach	The face-to-face approach used for these activities resulted in parents being unable to participate if they were away from home (38).
		Continuity	Short-term services do not guarantee a long-term sustainable effect (32, 41).
	Parents	Space	As this participant’s parents were working away from home, some activities were not available to them following space constraints (39, 40).
		Insufficient awareness of social service activities	Some participants’ family members had insufficient awareness of social workers’ responsibilities and were reluctant to inform social workers or aid in regulating parent–child relationships when issues arose (42).
Facilitator		Parent–child relationship	“I can actually understand how hard my dad works and I miss him, but he cannot be with me all the time and I cannot talk to him if I need something… Now it’s different, every time I talk to my dad by video, he asks me about the funny things that happened at school and I am willing to tell him” [(39), pp. 32–33]. “I talked to my mum and dad on the phone today and I showed them the handmade notebook I made. Mum and dad were especially happy when they saw it… Mum’s eyes were tearing up and I missed my mum too and did not want her to cry. I told my mum I would be good and not let my mum and dad worry” [(40), p. 39]. “I started expecting my parents to call me every day and I would get anxious if they called late” [(42), p. 19].

		ULBC self-awareness	Participants began to affirm themselves and recognise their strengths They describe themselves as helpful, good-tempered, considerate, good at crafts, and serious about their studies [(40), p. 39].
	ULBC self-confidence and interpersonal relationships	“I used to think that people did not like me and that other children had mothers and I did not, so I did not want to play with them. But at the last event, everyone complimented me on… Maybe I am not worse than others except that I do not have a mum. Now I have a few good friends in my class too and it feels so good to play together every day!” [(39), p. 32].
	Activity design	Interpersonal interaction	The experimental group had lower interpersonal health problem dimension and social avoidance scores than the control group post-intervention (32). After four sessions of group sandplay, the six students felt a sense of identification with each other and a sense of kinship (37). Up to 80% of the participants felt that they had a more open, easy-going personality and were happier after participating in the activity (44). Following the group activities, the participants became actively involved in the activities and voluntarily expressed their views while building good relationships with other members [(41), p. 37].
		General mental health	After the art therapy psychological intervention, 65 participants had significantly improved overall mental health, specifically anxiety regarding people, loneliness, and impulsivity (35). “My child has activities to participate in and peers to keep him company… He also learns new things and becomes sunny” [(43), p. 47]. “After four activities, I could clearly feel the change in her. She started to greet me, volunteered to talk to me about her academic life, and her academic performance improved greatly.” [(44), p. 46].
Behavioral deviance		After 12 weeks of DMT intervention, the experimental group denoted *p* < 0.05 for antisocial and neurotic behaviors and *p* < 0.05 for overall scores. All three results indicated significant differences between the experimental and control groups on the post-test data (33).
		Internet addiction	After five weeks of intervention, the experimental group spent an average of three hours less online per day compared to the control group. The experimental group had a significantly lower internet addiction total score than the control group (32).
		Psychological resilience	The participants discovered their strengths following group participation and began to feel self-confident [(41), p. 34]. They realised it was appropriate to seek assistance from those around them when encountering challenges [(41), p. 36].

#### Social workers

Social workers are the key catalysts of ULBC mental health services. Therefore, their expertise and practical experience directly affect activity progress and effectiveness. Six studies (39–41, 43–45) highlighted social workers as one of the main barriers to smooth activity progress. Reportedly, social workers were mainly university students (33, 34, 36, 38–44), local school teachers (32, 37), or library researchers (35). Only two of the studies received government funding (32, 37). This non-government funded activity was likely to have resulted in a rigorous evaluation and investigation of ULBC mental health activities owing to inadequate funding, effort, and staff. Song (43) documented the emergence of barriers in staff division and activity coordination when multi-format activities are available.

#### Activities

Several challenges have arisen in the implementation process, implementation approach, continuity of these mental health activities. Because multi-format activities encompass various group activities, coordinating and harmonizing the time and opinions of participants is more challenging, and individual opinions and issues may be overlooked (43). Furthermore, the uncontrollable outdoor environment hindered some outdoor activities (44). Additionally, Ge et al. (32) demonstrated that the participants became excessively involved in the psychodrama and found it challenging to calm down for a short period. Accordingly, the researchers suggested that the duration of ULBC psychodrama intervention should be extended to allow the participants to recover from the performance.

Implementation approach was another barrier to activity effectiveness. Based on the included studies, most of the activities were conducted face-to-face. Only one study increased parent–child communication using video calls (39). Nonetheless, the face-to-face approach proved challenging for most ULBC. The most significant challenge was their separation from their parents.

Ge et al. (32) and Wu (41) were concerned about the sustainability of the effects of the activities. Brief reunions in face-to-face parent–child interactions might obscure the parent–child relationship with idealisation. Therefore, the extent to which the effects of short-term activities are sustainable in the long-term requires further investigation.

#### Parents

Two studies reported that parents being in a different location resulted in the inability to engage with the service face-to-face (39, 40). Thus, Sun (39) and Yang (40) could only arrange for the participants and parents to interact via telephone and email. Furthermore, Zhang (42) demonstrated that the participants encountered barriers at the beginning of the ULBC family service activities given the participants’ family members’ lack of awareness about social workers’ work. This situation might have been due to the traditional Chinese belief where issues between family members cannot be revealed to outsiders. Therefore, the family members might have been reluctant to inform social workers or participate in reconciliation when parent–child relationships encountered issues.

However, parents are also important facilitators in the activities. Four studies (38–40, 42) demonstrated that parent–child interactions significantly improved the relationship between ULBC and their parents. Jiang (38) organised a group of six parent-ULBC families to play parent–child interaction games together. The other three studies involved social workers accessing ULBC families to communicate and guide their parent–child interactions. These studies suggested that the ULBC perceived and accepted parent–child interactions better by communicating with their parents. The ULBC begin to understand the hardships of their parents working outside the home and being unable to see them at home. Thus, the ULBC were willing to communicate voluntarily with their parents.

The parents also observed changes in the ULBC. Parents mentioned in interviews that ULBC became proactive in contacting parents (39), as well as proactively seeking help from parents when in trouble (40).

Parent–child interactions also influenced ULBC self-awareness (40) as they began to affirm themselves and recognise their own strengths (40). Additionally, parent–child interactions improved ULBC self-confidence and interpersonal relationships (39, 41, 42).

#### Activity design

The included studies suggested that group events, such as sandplay (37), psychodrama (32), activities (36, 41, 43, 44), and parent–child activities (38–40, 42), improved ULBC interpersonal relationships. Drawing (35) and multi-format activities (43, 44) significantly improved ULBC general mental health. Furthermore, DMT significantly improved ULBC behavioral deviance (33), while the psychodrama pairs significantly reduced the time spent online and improved Internet addiction (32). Moreover, the group activities improved ULBC self-identity, sense of purpose, and social competence, thus increasing psychological resilience (36, 41).

## Discussion

This review systematically evaluated the types of service delivery activities for ULBC mental health, their effectiveness, their implementation barriers and facilitators. Of the 14 studies reviewed in this review, there were nine group psychological activities, three individual family activities and two multi-format activities. All studies reported significantly improved ULBC mental health status after the activities.

The review suggests that for most of the social workers in the implementation of mental health activities for ULBC were all university students. While university students may possess some expertise in mental health and exhibit enthusiasm to serve, they are typically in the early stages of their academic training, which limits their ability to fully understand and apply the experience and expertise required to address complex mental health issues. Consequently, the depth of mental health activities implementation may be compromised.

Social work is a rapidly developing and emerging field in mainland China (46). Social workers play a crucial role in the implementation of mental health activities, as their professional knowledge and practical experience significantly influence the effectiveness of the activities. However, many social workers face challenges such as insufficient training, limited resources, and high work pressure, which can hinder the utilisation of their skills and, consequently, impact the effectiveness of their activities (47). Therefore, the Chinese government and funding agencies should consider providing ongoing training in professional competencies to enable social workers to more effectively assist those in need.

This study found that most of these mental health activities used face-to-face, with only one study (39) using video calls to enhance parent–child communication. The face-to-face approach poses significant challenges for many ULBC families, as they often experience the greatest difficulty with parent–child separation. Additionally, this approach limits participation, as only a small number of participants can be served at a time, and some may be unable to attend due to constraints such as time, transportation, or cost. Therefore, future studies could explore the use of online technologies to create virtual participation opportunities for parents, thereby improving accessibility and engagement in ULBC mental health activities.

This review indicates that parents play a dual role as both challenges and facilitators in the activity implementation, and their perceptions and attitudes significantly influence its success. Under the influence of the “keeping the shame of the family in the family” concept, some parents exhibited uncooperative attitudes during interactions, leading to a lack of motivation and interest in ULBC, and even hindering the smooth implementation of the activities (42). In contrast, cooperative parents were more willing to invest time and energy in communicating with ULBC, thereby achieving, or even exceeding, the activities’ expected outcomes. Therefore, future studies should explore strategies to enhance parents’ awareness and motivation for mental health activities, thereby fostering more effective family participation. Additionally, research should focus on strengthening the trust between social workers and parents to ensure the smooth implementation of activities with family support.

Finally, while most of the included studies focused on the explicit and implicit left-behind characteristics of ULBC; however, they did not provide more targeted mental health activities for these different characteristics. Therefore, future studies should consider developing more comprehensive and tailored mental health activities based on the various left-behind characteristics of ULBC. Such studies will provide more specific practical guidance for psychologists, sociologists, educators, and policymakers to optimise activity design and more effectively promote the mental health development of ULBC.

### Strengths and limitations

To the best of the authors’ knowledge, this study is the first systematic review of the implementation of mental health activities for ULBC. Most of the included studies were qualitative, resulting in descriptive data. The richness of these data enabled researchers to gain a deeper understanding of the influences of activities on the mental health of ULBC and to further analyse and compare these influences. This review provides valuable evidence for mental health activities targeting ULBC, offering important insights that can guide the design and implementation of service activities, as well as the planning and development of related policies.

This systematic review also encountered some limitations, such as the measurement scales used. The included studies assessed ULBC mental health of ULBC using different measurement scales based on the differing mental health issues. For example, Wu (41) examined ULBC social resilience pre-intervention using a questionnaire but assessed the intervention outcome using interviews. Furthermore, Jiang (38) measured the parent–child relationship pre-activity using the self-administered parent–child relationship questionnaire and assessed the parent–child relationship post-activity using the Chinese adaptation of the PCRT. Additionally, Zhang (37) screened ULBC with poor interpersonal relationships for the intervention using the Chinese adaptation of the IRRS, and assessed the intervention outcomes using post-intervention observations of daily performance and classroom teacher and peer interviews. The researchers did not provide rationales for the inconsistent pre-post assessment approaches used, which might have affected the reliability and validity of their results. Therefore, future research should provide more detailed methodological information to improve validity and reliability. Thus, the instruments could not be compared significantly.

Some of the included studies had a notably small sample sizes (only one person) (39, 40, 42) and did not provide a reason for selecting only one participant. The small sample size may have hindered the study’s ability to provide a comprehensive overview of ULBC’s mental health activities, thereby reducing the robustness of its findings. Additionally, a small sample size limits the study’s representativeness of the broader ULBC population, potentially restricting the applicability of its findings.

## Data Availability

The original contributions presented in the study are included in the article/supplementary material, further inquiries can be directed to the corresponding author.
